# Vaccinomics Approach for Designing Potential Peptide Vaccine by Targeting *Shigella* spp. Serine Protease Autotransporter Subfamily Protein SigA

**DOI:** 10.1155/2017/6412353

**Published:** 2017-09-07

**Authors:** Arafat Rahman Oany, Tahmina Pervin, Mamun Mia, Motaher Hossain, Mohammad Shahnaij, Shahin Mahmud, K. M. Kaderi Kibria

**Affiliations:** ^1^Department of Biotechnology and Genetic Engineering, Faculty of Life Science, Mawlana Bhashani Science and Technology University, Tangail, Bangladesh; ^2^Biotechnology and Genetic Engineering Discipline, Life Science School, Khulna University, Khulna, Bangladesh; ^3^Department of Biochemistry and Molecular Biology, University of Dhaka, Dhaka, Bangladesh; ^4^Enteric and Food Microbiology Laboratory, International Centre for Diarrhoeal Disease Research Bangladesh (icddr,b), Dhaka, Bangladesh

## Abstract

Shigellosis, a bacillary dysentery, is closely associated with diarrhoea in human and causes infection of 165 million people worldwide per year. Casein-degrading serine protease autotransporter of enterobacteriaceae (SPATE) subfamily protein SigA, an outer membrane protein, exerts both cytopathic and enterotoxic effects especially cytopathic to human epithelial cell type-2 (HEp-2) and is shown to be highly immunogenic. In the present study, we have tried to impose the vaccinomics approach for designing a common peptide vaccine candidate against the immunogenic SigA of *Shigella* spp. At first, 44 SigA proteins from different variants of *S. flexneri*, *S. dysenteriae*, *S. boydii*, and *S. sonnei* were assessed to find the most antigenic protein. We retrieved 12 peptides based on the highest score for human leukocyte antigen (HLA) supertypes analysed by NetCTL. Initially, these peptides were assessed for the affinity with MHC class I and class II alleles, and four potential core epitopes VTARAGLGY, FHTVTVNTL, HTTWTLTGY, and IELAGTLTL were selected. From these, FHTVTVNTL and IELAGTLTL peptides were shown to have 100% conservancy. Finally, IELAGTLTL was shown to have the highest population coverage (83.86%) among the whole world population. In vivo study of the proposed epitope might contribute to the development of functional and unique widespread vaccine, which might be an operative alleyway to thwart dysentery from the world.

## 1. Background

*Shigella* is a Gram-negative, facultative anaerobic, nonmotile, nonspore forming, and rod-shaped true bacteria closely related to *Salmonella* and *Escherichia coli*. The resulting infection by this organism called shigellosis, also known as bacillary dysentery or Marlow syndrome, is most typically associated with diarrhoea and other gastrointestinal symptoms in humans. This pathogen is usually found in water that is contaminated with human feces within the setting of poor hygiene among kids of underneath 5 years old and is transmitted via the fecal-oral route. The infection will occur even if there is just a bodily function of only ten to one hundred microorganisms [1]. In each year, 165 million cases of *Shigella* infection are accounted worldwide, of that, 163 million take place in developing countries and ultimately result in millions of death [2]. Bangladesh has got the top rates of shigellosis according to the recent Global Enteric Multicenter Study (GEMS) in Asia. The output of this study has revealed that the *Shigella* is the third leading reason behind diarrhoea in children [3, 4].

*Shigella* species are usually classified into four serogroups: *S. dysenteriae* (12 serotypes), *S. flexneri* (6 serotypes), *S. boydii* (18 serotypes), and *S. sonnei* (one serotype) based on the biochemical properties and group-specific O antigens within the outer portion of the semipermeable membrane. *S. dysenteriae*, *S. flexneri*, and *S. boydii* are physiologically similar in distinction to *S. sonnei*. Among them, *S. flexneri* is the most frequently isolated species globally and accounts for 60% of cases in the unindustrialized countries; *S. sonnei* causes 77% of cases in the industrialized countries [1].

The underlying therapeutic challenge to manage *Shigella* is its accrued resistance to most often used antibiotics like ampicillin, tetracycline, streptomycin, nalidixic acid, and sulfamethoxazole-trimethoprim [5]. Earlier, ciprofloxacin, a third-generation fluoroquinolone antibiotic, has been used effectively for the treatment of bacillary dysentery [6]. However, this antibiotic is no longer helpful for the treatment of bacillary dysentery in south Asian countries together with Bangladesh, because of the dissemination of fluoroquinolone-resistant *variety* and its equivalent clones across the countries [7, 8]. Hence, it is essential to find a sustainable approach like vaccinomics, which can elicit long-term and consistent immunological responses to fight against *Shigella*.

SigA is annotated in the she pathogenicity island of *Shigella*, encoding SigA protein which belongs to the serine protease autotransporter of enterobacteriaceae (SPATE) subgroup proteins. The autotransporter proteins of Gram-negative bacteria exhibit an N-terminal signal sequence, required for secretion across the inner membrane, and a C-terminal domain that forms an amphipathic *β*-barrel pore that allows passage of the functional domain across the outer membrane. This type of exporter proteins either remains attached to the cell surface or is released from the cell by proteolytic cleavage [9]. SigA is a multifunctional protein, able to degrade casein with cytotoxic and enterotoxic effects. Moreover, SigA is cytopathic for human epithelial type-2 (HEp-2) cells, causing morphological changes and loss of integrity of the cell monolayers, important for the pathologic process of *Shigella* [10]. The position of SigA in the chromosome made them less vulnerable to loss compare to the other virulence factors harbouring within the plasmid, and more exposure to the immune cells occurred by this secreted toxin [11]. Most importantly, this protein has been shown to be immunogenic following infection with *Shigella* [10]. The generalized modules of membrane antigen- (GMMA-) based outer membrane proteins including SigA were also shown to be highly immunogenic [12], which prompted us to target SigA as one of the best vaccine candidates and to design potential peptide vaccine covering all the *Shigella* spp. and most of the regions of the world.

Epitope-based immunizing agents are often an inexpensive choice to thwart enteric *Shigella* infection. The identification of specific epitopes derived from infectious pathogens has considerably advanced the event of epitope-based vaccines (EVs). Higher understanding of the molecular basis of substance recognition and human leukocyte antigen- (HLA-) binding motifs has resulted in the advancement of rationally designed vaccines that solely depends on algorithms predicting the peptide's binding to human HLA. The traditional process for the development of a vaccine is very complex compared to that of the epitope-based vaccine, and additionally, it is chemically stable, more specific, and free of any infectious or oncogenic potential hazard [13]. However, the invention of a wet laboratory-based candidate epitope is expensive and laborious that requires varied medicine experiments in the laboratory for the ultimate choice of epitopes. Hence, the interest for predicting epitopes by computational strategies, alternate in silico approaches among researchers, is growing bit by bit with reduced efforts.

Vaccinomics is the application of integrated knowledge from different disciplines including immunogenetics and immunogenomics to develop candidate next-generation vaccine and understand its immune response [14]. Currently, various vaccinomics databases are accessible for identification of distinctive B lymphocyte epitopes and HLA ligands with high sensitivity and specificity [15–17]. The vaccinomics approach has already proven its potency in identifying the conserved epitope in the case of human immunodeficiency virus [18], multiple sclerosis [19], tuberculosis [20], and malaria [21] with desired results. In our study, we have applied vaccinomics approaches for the screening of potentially conserved epitopes by targeting protein SigA.

## 2. Methods

**Figure 1:**
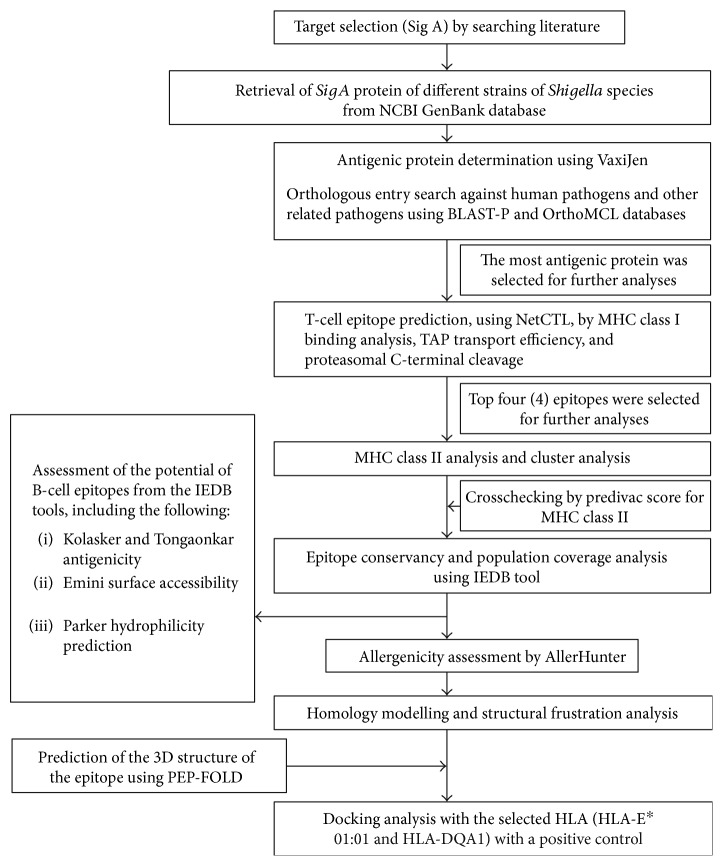
Flow diagram of the methodology.

The flow chart summarizing the protocols for the complete epitope prediction is illustrated in Figure 1.

### 2.1. Sequence Retrieval and Antigenic Protein Determination

The SigA protein sequences of different strains of *Shigella* species were retrieved from the NCBI GenBank [22] database and analysed in the VaxiJen v2.0 [23] server for the determination of the most potent antigenic protein. Additionally, the target protein was crosschecked against human pathogens and other similar pathogens to ensure the orthologous entry by using BLAST-P [24] and OrthoMCL [25] databases [26].

### 2.2. T-Cell Epitope Prediction and Affinity with MHC

The epitope prediction for the respective protein and their affinity score with MHC class I and class II allele was measured following previously used approach [27, 28]. Concisely, the NetCTL v1.2 server [29] was used for predicting potential cytotoxic T-lymphocyte (CTL) epitopes from the most antigenic protein. A combined algorithms including MHC-I binding, transporter of antigenic peptide (TAP) transport efficiency, and proteasomal C-terminal cleavage prediction were employed for the T-cell epitope prediction. The epitope with the highest score for 12 MHC class I supertypes was selected.

T Cell Epitope Prediction Tools from Immune Epitope Database and Analysis Resource (IEDB-AR) were used for the prediction of affinity with MHC class I [30] and MHC class II [31, 32]. The stabilized matrix method (SMM) was used to calculate the half-maximal inhibitory concentration (IC_50_) of peptide binding to MHC class I with a preselected 9.0-mer epitope. The peptides were also assessed for HLA I binding affinity by the software, EPISOFT. For the analyses of MHC class II binding, the IEDB-recommended method was used for the specific HLA-DP, HLA-DQ, and HLA-DR loci. Fifteen-mer epitopes were designed for MHC class II binding analysis considering the preselected 9-mer epitope and its conserved region in the *Shigella* strains. For the MHC class I and MHC class II alleles, the epitopes consisting IC_50_ < 250 nM and IC_50_ < 100 nM, respectively, were selected for further analysis. The MHC class II binding prediction tool PREDIVAC was also used to assess their affinity with HLA_DRB_1.

### 2.3. Cluster Analysis of the MHC Restricted Alleles

Furthermore, the MHCcluster v2.0 server [33] was used for the identification of cluster of MHC restricted allele with appropriate peptides to further strengthen our prediction. This is the additional crosscheck of the predicted MHC restricted allele analysis from the IEDB analysis resources. The output from this server is a static heat map and a graphical tree for describing the functional relationship between peptides and HLAs.

### 2.4. Epitope Conservancy and Population Coverage Analyses

Epitope conservancy of the candidate epitopes was examined using a web-based epitope conservancy tool available in IEDB analysis resource [34]. The conservancy level of each potential epitope was calculated by considering identities in all SigA protein sequences of different strains retrieved from the database. Multiple sequence alignment (MSA) was employed to understand the positions of the epitopes within the sequences. As SPATE family is very much specific for the enterobacteria, specifically, *E. coli* and *Shigella*, we also include two *E. coli* sequences (gi|693049347| and gi|699401135|) along with those of four species of *Shigella* for MSA construction. The Jalview (http://www.jalview.org/) tool was used for this analysis. The conservancy of the selected peptides was also substantiated by the Protein Variability Software (PVS) [35]. Population coverage for the epitope was assessed by the IEDB population coverage calculation tool [36]. The combined score for MHC classes I and II was assessed for the analysis of the population coverage.

### 2.5. Homology Modelling and Structural Frustration Analysis

A homology model of the conserved region was obtained by MODELLER v9 [37], and the predicted model was assessed by the PROCHECK [38, 39] server. For the disorder prediction among the amino acid sequences, DISOPRED v3 [40] was used. The protein frustratometer server [41] was employed for the detection of the stability and energy differences of the 3D structure of the protein.

### 2.6. Molecular Docking Analysis and HLA Allele Interaction

Docking studies were also performed using the best possible epitope following the strategy used in previous studies [27, 28]. AutoDock Vina [42] was used for the docking analysis. In our study, we have selected the HLA-E∗01:01 molecule as a candidate for MHC class I and the HLA-DQA1 as a candidate for MHC class II for docking analysis because they are the available hits in the Protein Data Bank (PDB) database. The PDB structure 2ESV, human cytomegalovirus complexes with T-cell receptors, VMAPRTLIL peptide, and 3PL6—structure of autoimmune TCR Hy.1B11 in complex with HLA-DQ1—were retrieved from the Research Collaboratory for Structural Bioinformatics (RCSB) protein database [43]. Then, the structures were simplified by using PyMOL (the PyMOL Molecular Graphics System, Version 1.5.0.4, Schrödinger, LLC) for the final docking purpose.

The PEP-FOLD server [44] was used for the conversion of the 3D structure of the epitope “IELAGTLTL” for MHC I and the epitope “KAIELAGTLTLTGTP” for the MHC II molecule in order to analyse the interaction with HLA alleles.

Finally, molecular docking was performed at the center of *X*: 77.8087, *Y*: −3.2264, and *Z*: −9.5769 and the dimensions (angstrom) of *X*: 31.4432, *Y*: 29.9517, and *Z*: 19.0455 for the MHC I molecules. For the MHC II molecules, docking was performed at the center of *X*: 38.5584, *Y*: 46.6132, and *Z*: −36.4392 and the dimensions (angstrom) of *X*: 34.8104, *Y*: 40.4401, and *Z*: 37.3366. Additionally, we have performed a control docking with the experimentally known peptide—MHC-bound complex. The PDB structure 2ESV, human cytomegalovirus complexes with T-cell receptors, and VMAPRTLIL peptide were used for this purpose. The gridline was used at the center of X: 77.3404, Y: −3.5159, and Z: −9.5829.

### 2.7. Allergenicity Investigation and B-Cell Epitope Prediction

The AllerHunter server [45] was used to predict the allergenicity of our proposed epitope for further securing the prediction, and the support vector machine (SVM) algorithm was used for the prediction within the server [46]. The predicted T-cell epitope (15-mer) was screened by IEDB-AR using a number of web-based tools for the suitability as the B-cell epitope [47–49].

## 3. Results

### 3.1. Analysis of the Retrieved Sequences and Their Antigenicity

A total of 44 SigA proteins from different variants of *S. flexneri*, *S. dysenteriae*, *S. boydii*, and *S. sonnei* were retrieved from the GenBank database (Table S1 in Supplementary Material available online at https://doi.org/10.1155/2017/6412353). Thereafter, analyses with the VaxiJen v2.0 server showed the protein with the accession number of gi|745767180| to have the highest antigenicity of 0.6699 (Table S1). This highly antigenic protein was further analysed to detect the highly immunogenic epitope. No significant entry was found in the orthologous entry search of our targeted protein.

### 3.2. T-Cell Epitope Identification

The NetCTLv1.2 server identified the T-cell epitopes, where the epitope prediction was confined to 12 MHC class I supertypes. Based on the combined score, the top twelve epitopes (Table 1) were listed for further analysis.

### 3.3. MHC Restriction and Cluster Analysis

**Figure 2:**
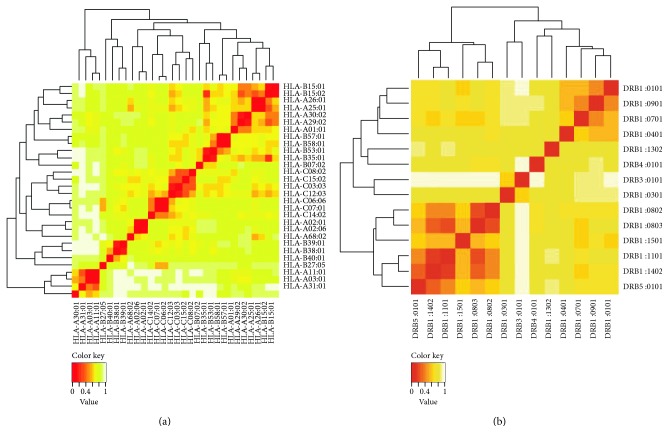
Cluster analysis of the HLA alleles for both MHC molecules through heat map representation. (a) Representing the cluster of the MHC-I. (b) Representing the cluster of MHC-II molecules. Epitopes are clustered on the basis of interaction with HLA and shown as red colour indicating strong interaction with appropriate annotation. Yellow zone indicates the weaker interaction. Here, all the available alleles are shown only.

IEDB analysis resource predicted both MHC class I and MHC class II restricted allele on the basis of the IC_50_ value. All the predicted epitopes in Table 1 were assessed for the MHC interaction analysis. Epitopes for the MHC class I alleles are presented in Table 2. The peptide IELAGTLTLT was predicted to have the highest number of MHC class I binding. This peptide was predicted to have the binding affinity with five MHC class I alleles including HLA-E∗01:01, HLA-B∗40:01, HLA-B∗15:02, HLA-C∗03:03, and HLA-C∗12:03. Furthermore, the interacted alleles were reassessed by cluster analysis and are shown in Figure 2(a), as a heat map, and in Figure S1A, as a dynamic tree. The peptides were reassessed by the EPISOPT software for the HLA I binding, and IELAGTLTL was found to have affinity with six HLA I alleles (Table S3). From this analysis, we selected top four peptides VTARAGLGY, FHTVTVNTL, HTTWTLTGY, and IELAGTLTL depending on the affinity with most MHC class I.

Epitopes for the MHC class II alleles are presented in Table 3. Depending on the IC_50_ values as well as on the number of MHC class II alleles, three 15-mer peptide candidates were selected. The peptides NSG**FHTVTVNTL**DAT, KA**IELAGTLTL**TGTP, and AAKS**YMSGNYKAF**LT were predicted to have high affinity with MHC-II allele, which can interact with 32, 29, and 24 MHC class II alleles. The data has been validated by another software PREDIVAC. The predivac scores of the two core peptides FHTVTVNTL and IELAGTLTL have been shown to be promising for their binding to HLA_DRB_1 (Table 3). Accumulating both MHC class I allele- and MHC class II allele-based analyses, we showed FHTVTVNTL and IELAGTLTL peptides to have the best score to be a vaccine potential.

### 3.4. Conservancy Analysis and Position of the Epitopes

**Figure 3:**
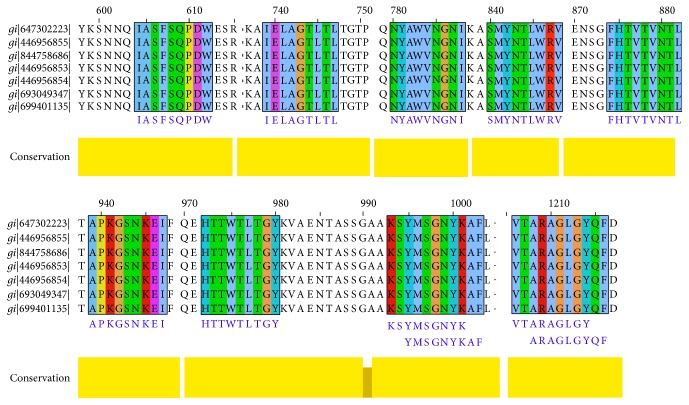
MSA-based location identification of the different epitopes within the SPATE proteins of *Shigella* and their homologue in *E. coli*. In this figure, gi|647302223|, gi|446956855|, gi|844758686|, and gi|446956853| represent the *S. flexneri*, *S. sonnei*, *S. boydii*, and *S. dysenteria*e, respectively. *E. coli* represented by gi|693049347| and gi|699401135|.

**Figure 4:**
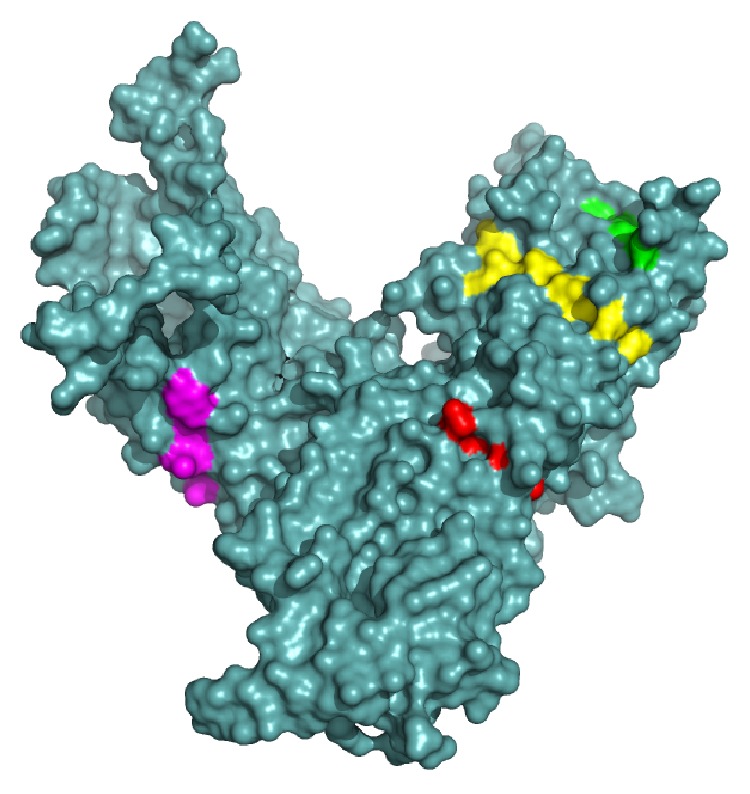
The three-dimensional model of SPATE subfamily protein SigA with the proposed epitopes VTARAGLGY (magenta), FHTVTVNTL (yellow), HTTWTLTGY (green), and IELAGTLTL (red). The superficial localities of the epitopes indicate their surface accessibility.

Conservancy of all the proposed epitopes was assessed by the IEDB conservancy analysis tool and is summarized in Table 4. FHTVTVNTL, IELAGTLTL, NYAWVNGNI, and SMYNTLWRV were shown to have 100% conserved regions across all the SigA proteins. The position of all the predicted epitopes is shown in a multiple sequence alignment of SigA proteins in Figure 3. Here, we used only our desired sequences for the proper annotation. So, from the most potential candidates, only two, that is, FHTVTVNTL and IELAGTLTL, were found to be fully conserved. The top four epitopes were shown within the protein in Figure 4. The conservancy of both of these peptides were crosschecked by PVS software, and it was found that they were located in the conserved region of the SigA protein (Figure S5). The epitopes are precisely positioned on the surface of the protein indicating that they would be accessible to the immune system, especially by B-cells.

### 3.5. Model Validation Structural Frustration Analysis

**Figure 5:**
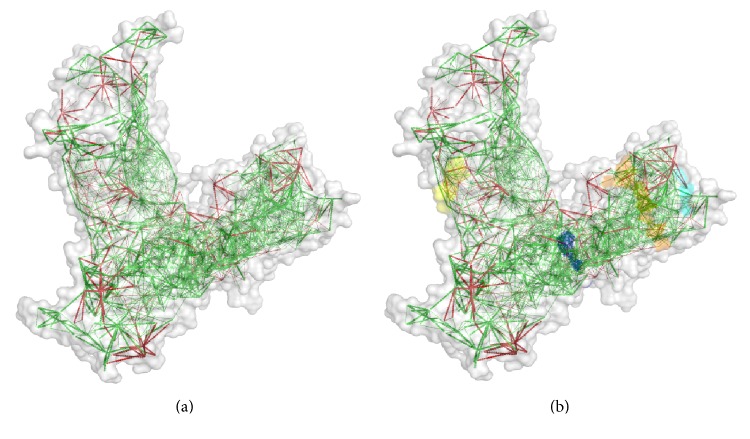
The configurational frustration index of the predicted model of the SigA. (a) This analysis detects the stability and energy differences of the 3D structure of the protein. Colours are in accordance with their frustration index. The red colour regions are highly frustrated and the green colour regions are not frustrated. The frustrated residues are able to change their identity and also displace the location in any favourable conditions. (b) The locations of our proposed epitopes are described by different colours. The epitopes HTTWTLTGY (cyan) and IELAGTLTL (blue) are well outside of the frustrated regions and securing their stability. On the other hand, the epitopes VTARAGLGY (yellow) and FHTVTVNTL (orange) are in the frustrated regions and unable to secure their stability.

MODELLER modelled the three-dimensional structure of the targeted protein through the best multiple template-based modelling approach. The validation of the model was measured by the PROCHECK server through the Ramachandran plot and is depicted in Figure S2, where 88.8% amino acid residues were found within the favoured region. Furthermore, the predicted model was also assessed for the frustration analysis and is depicted in Figure 5. The DISOPRED server likewise assessed the disorder of the protein sequences in order to get an understanding about the disorder among the targeted sequences, which is shown in Figure S3.

### 3.6. Population Coverage Analysis

**Figure 6:**
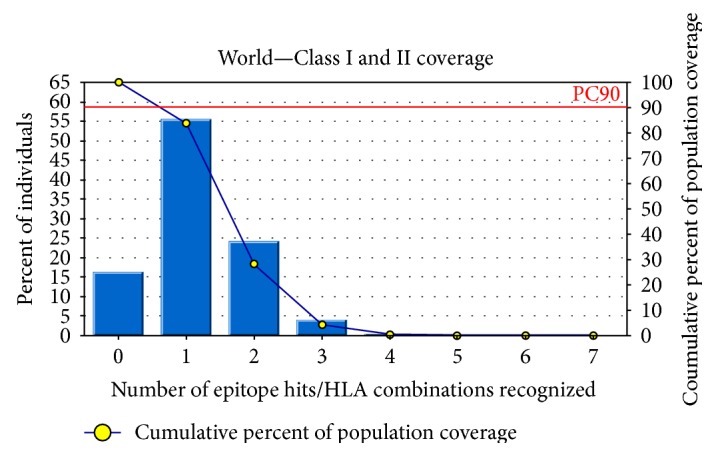
Population coverage analysis for the top predicted epitope based on the HLA interaction. Here, the whole world populations are assessed for the proposed epitope. The combined prediction for both of the MHC has been shown. Here, the number 1 bar for all the analyses represents out-predicted epitope. Notes: in the graphs, the line (-o-) represents the cumulative percentage of population coverage of the epitopes; the bars represent the population coverage for each epitope.

IEDB analysis resource predicted both MHC class I- and MHC class II-based coverage of the selected epitopes for the world population to assess the feasibility of being a potential vaccine candidate. The combined prediction was also assessed. The epitope “IELAGTLTLT” has the highest population coverage of 83.86% for the whole world population (shown graphically in Figure 6); however, another potential epitope “FHTVTVNTL” was shown to have 50.61% population coverage (Table S2).

### 3.7. Molecular Docking Analysis

**Figure 7:**
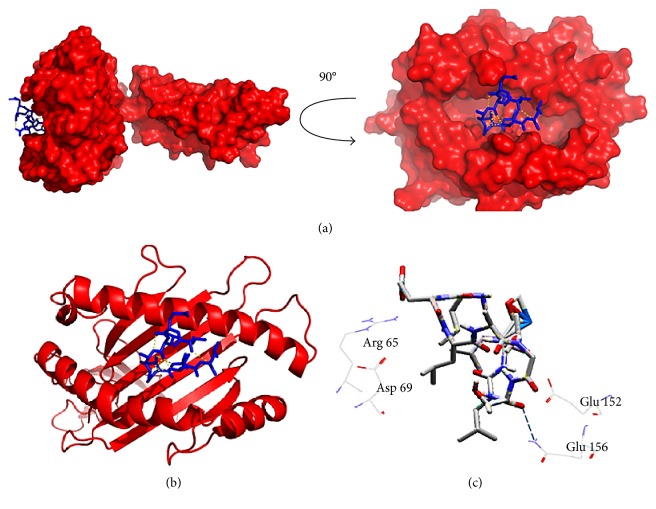
Docking analysis of the predicted epitope IELAGTLTL and HLA-E allele. (a) Representing the oriented view of the interaction and assuring the perfect binding. (b) Representing the cartoon view. (c) Embodying the interacted residues with the peptide.

**Figure 8:**
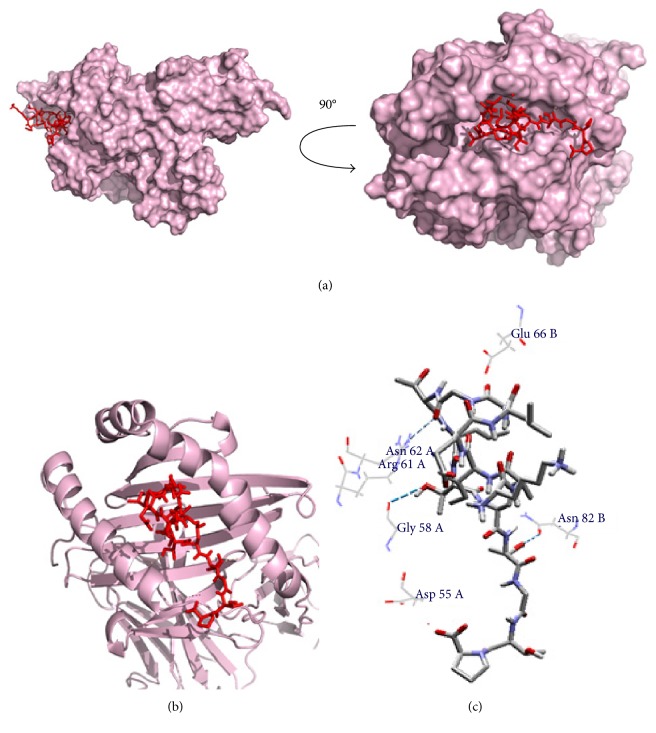
Docking analysis of the predicted epitope KAIELAGTLTLTGTP and HLA-DQA1 allele. (a) Representing the oriented view of the interaction and assuring the perfect binding. (b) Representing the cartoon view. (c) Embodying the interacted residues with the peptide.

The core epitope (IELAGTLTL) with 9.0 mer and its 15-mer extension (KAIELAGTLTLTGTP) were bound in the groove of the HLA-E∗01:01 and HLA-DQA1 with an energy of −7.8 and −9.7 kcal/mol, respectively. AutoDock Vina generated different poses of the docked peptide, and the best one was picked for the final calculation at an RMSD (root-mean-square deviation) value of 0.0. The docking interface was visualized with the PyMOL Molecular Graphics System. The 9.0-mer epitope interacted with Arg-61, Asn-62, and Glu-152 through steric interaction and formed hydrogen bonding with the Glu-156 amino acid residues. On the other hand, the 15-mer epitope interacted with Asp-55 through electrostatic interaction and Glu-66 through steric interaction and formed hydrogen bonding with the Gly-58, Arg-61, Asn-62, and Asn-82 amino acid residues. The docking output and the interacted residues are shown in Figures 7 and 8 with different orientations. Furthermore, the control docking energy was found to be −6.8 kcal/mol and is illustrated in Figure S4.

### 3.8. Allergenicity Analysis

The AllerHunter web server predicted the sequence-based allergenicity calculation very precisely. The allergenicity of the queried core epitope (IELAGTLTLT) was 0.05 (sensitivity = 98.40%, specificity = 27.4%), and the allergenicity of the 15-mer epitope (KAIELAGTLTLTGTP) was 0.05 (sensitivity = 98.4%, specificity = 27.0%).

### 3.9. B-Cell Epitope Prediction

**Figure 9:**
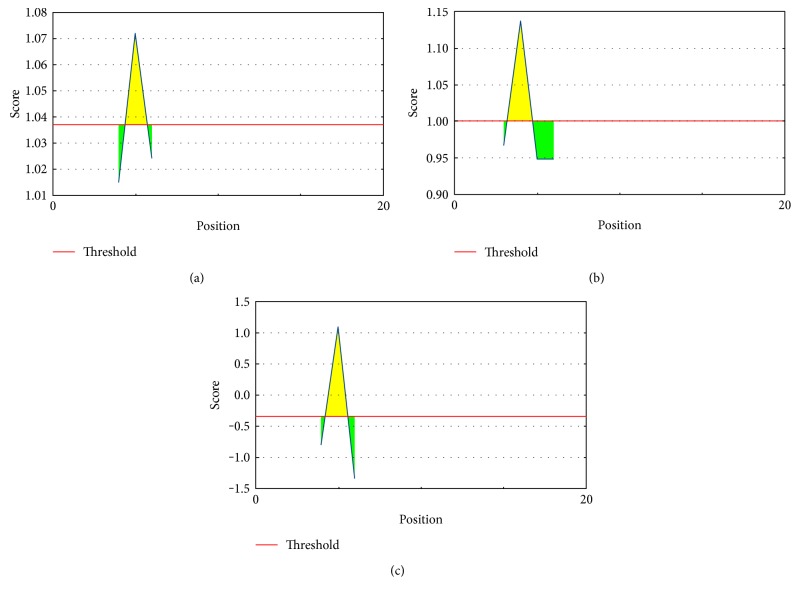
B-cell epitope prediction. (a) Kolaskar and Tongaonkar antigenicity prediction of the proposed epitope with a threshold value of 1.037. (b) Emini surface accessibility prediction of the proposed epitope, with a threshold value of 1.0. (c) Parker hydrophilicity prediction of the epitope, with a threshold of −0.352. Notes: the *x*-axis and *y*-axis represent the sequence position and antigenic propensity, respectively. The regions above the threshold are antigenic (desired), shown in yellow.

B-cell epitope prediction was obtained for the peptide KAIELAGTLTLTGTP (15 mer) through the sequence-based approaches, and values are anticipated with different parameters, ranging from −0.6464 to 1.137. These values are the different propensity scores and predicted with a threshold ranging from −0.352 to 1.037 (Figure 9()). The Kolaskar and Tongaonkar antigenicity scale was employed for evaluating the antigenic property of the peptide with a maximum of 1.072. The antigenic plot is showed in Figure 9(a). Peptide surface accessibility is another important benchmark to meet up the criteria of a potential B-cell epitope. Henceforth, Emini surface accessibility prediction was employed, with a maximum propensity score of 1.137 (Figure 9(b)). To reinforce our provision for the prediction of the epitope to elicit B-cell response, the Parker hydrophilicity prediction was also employed with a maximum score of 1.086 and is depicted in Figure 9(c).

## 4. Discussion

Enteric infections are the foremost cause of sickness and impermanence throughout the world, and only the *Shigella* infections resulted in over a million deaths annually [2]. The ever rising multidrug-resistant (MDR) strains of the *Shigella* bacteria area unit are another international concern for the researchers to search out a brand new resolution for preventing the deaths [50, 51]. Recently, there are several studies that focus on the development of the vaccine against *Shigella* and continue in the clinical trial. Most of them use attenuated and inactivated preparation of the bacteria for eliciting immune responses which has some potential escape risk [52–54]. In this study, we have tried to find out alternatives to treat this global burden through vaccinomics approaches and targeting the immunogenic and toxic protein SigA. The sequences of different strains of *Shigella* showed that there is a little island of conserved sequence throughout the species [55], and we have focused on that target for designing the vaccine candidate. The orthologous entry search of our targeted protein revealed no significant similarity with human pathogens and other closely related pathogens. These results further strengthen our prediction through confirming no cross immunity.

In recent time, most of the vaccines are grounded on B-cell immunity; vaccines based on a T-cell epitope have been invigorated lately. This is often as a result of body substance response from memory B-cells which may be overawed basically by matter drift as time goes on, whereas cell-mediated immunity repeatedly delivers long-run immunity [56, 57]. As a consequence, a T-lymphocyte epitope elicits a robust and distinctive immune response through the cytotoxic lymphocyte- (CTL-) mediated pathway and impedes the spreading of the infectious agents by the CTL through recognizing and killing the infected cells or by secreting specific cytokines [58].

The epitopes VTARAGLGY, FHTVTVNTL, HTTWTLTGY, and IELAGTLTL are primarily selected for the designing of vaccine from the initial analysis depending on the affinity with MHC class I and additionally confirmed their presence along with those of the ancestral homologue in *E. coli* (Figure 2). Finally, through substantiation with different parameters, the core epitopes IELAGTLTL and FHTVTVNTL (in 15.0-mer form, KAIELAGTLTLTGTP and NSGFHTVTVNTLDAT, resp.) were found to be the most potential and highly interacting HLA candidates for MHC class II molecule. Furthermore, we have used pSORTb to predict the subcellular localization of SigA and found that there is a score of 5.87 for localization in the outer membrane and another score of 4.13 for extracellular localization. The result was quite similar with that for the localization of other SPATE proteins in the bacterial cell surface as well as in secreted forms.

The three-dimensional model built through MODELLER and validated by the Ramachandran plot with an acceptable range resulted in the display of the perfect position of the epitope on the surface of the structure. As the epitope was found on the surface (Figure 4) of the model, it would increase the possibility to interact with the immune system earlier. Furthermore, the analysis from the DISOPRED and frustration analysis servers strengthen our prediction, though there are no disorder and energy frustration in the epitope region of the sequences and model, respectively (Figure 5 and Figure S3).

To get the acceptability, vaccine candidates must have wider population coverage. This is very much important before designing. In our analysis, we have found that our proposed epitope IELAGTLTL had combined population coverage of 83.86%, whereas the other most potential candidate FHTVTVNTL had combined population coverage of 50.61%. This output revealed that the proposed epitopes would have wider coverage *in vitro*.

Molecular docking upkeeps the prediction with a higher docking score and the perfectly oriented interactions between the both MHC and the predicted 9.0-mer and 15-mer epitopes. Additionally, comparative analysis with the experimentally known peptide—MHC complex—has also revealed the precision of our prediction through the similar binding energy and interacted residues. Another significant finding is the conservancy result. Through analysis of the whole retrieved sequences, it was found that our predicted epitopes have a 100% conservancy and hopefully they would be potential candidates for treating all of the *Shigella* spp. Our proposed epitopes are nonallergenic in nature according to the FAO/WHO allergenicity evaluation scheme.

Finally, the core epitope “IELAGTLTL” was also found to be more potential B-cell epitope candidates that were proposed through the sequence-based approaches including the Kolaskar and Tongaonkar antigenicity scale, Emini surface accessibility prediction, and Parker hydrophilicity prediction. From the overhead analysis, we envisage that our suggested epitope would also elicit an immune response *in vitro*.

## 5. Conclusion

The improved knowledge about antigen recognition at molecular level led us to the development of rationally designed peptide vaccines. The idea of peptide vaccines is based on detecting and chemical synthesis of immunodominant B-cell and T-cell epitopes capable of evoking specific immune responses. In this study, we used different computational tools to identify potential epitope targets against *Shigella* which will help to decrease the cost and time of wet lab experiments more successfully. Our bioinformatic analyses speculate that the selected part of the outer membrane and highly immunogenic protein, SigA, is a potential candidate for a peptide vaccine. It might also contribute to the reduction in the SigA-mediated pathogenicity to the host. However, further wet lab validation is necessary to confirm the efficiency of our identified peptide sequence as an epitope vaccine against *Shigella*.

## Supplementary Material

Table S1: Protein sequences retrieved from NCBI GenBank database for the analysis with appropriate accession numbers and antigenic score. Table S2: Population coverage analysis of the epitopes. Here the combined score for both MHC molecules have been represented. Table S3: The HLA I binding profile analyses of the selected peptides by EPISOPT. Figure S1: Dynamic interaction analysis of the clustered HLA alleles through tree illustration. Figure1A represents the MHC-I and Figure1B represents the MHC-II interaction. Here, HLA molecules are clustered on the basis of their preference for interaction with appropriate peptides. Figure S2: Ramachandran plot of the predicted model, which shows that most of the residues are in the allowed region of the plot, proving the validity of the model. Figure S3: Disorder prediction of the amino acid sequences of SigA. Here, our proposed epitopes localized outside of the disordered regions (15-35 and 520-530) and securing their potentiality for being an effective vaccine candidate. Notes: Amino acids in the input sequence are considered disordered when the blue line is above the gray dashed line, that is, when the confidence score is 0.5. The orange line shows the confidence score of the disordered protein-binding residue predictions. Figure S4: Control docking analysis of the epitope VMAPRTLIL and HLA-E allele. Figure S4A represents the oriented view of the interaction and assuring the perfect binding. Figure S4B represents the cartoon view and the Figure S4C embodies the interacted residues with the peptide. Figure S5: Variability plot for the 44 SigA protein alignment. The analysis was performed by using the PVS (protein Variability Software). The selected peptides were observed in the conserved region highlighted in the table.

## Figures and Tables

**Table 1:** T-cell epitopes of SigA protein predicted by the NetCTL server on the basis of the combined score. Here, epitopes for all the 12 different HLA supertypes have been presented.

Supertypes	Epitope	Start position	Combined score
A1	VTARAGLGY	645	2.8475
A2	SMYNTLWRV	279	1.4558
A3	KSYMSGNYK	432	1.5812
A24	NYAWVNGNI	219	1.6813
A26	HTTWTLTGY	411	2.0947
B7	APKGSNKEI	378	1.1981
B8	YMSGNYKAF	434	1.4879
B27	ARAGLGYQF	647	1.7245
B39	FHTVTVNTL	313	2.6855
B44	IELAGTLTL	178	1.9586
B58	IASFSQPDW	43	1.9421
B62	YMSGNYKAF	434	1.4814

**Table 2:** Epitopes for CD8^+^ T-cell along with their interacting MHC class I alleles with affinity < 250 nM.

Epitope	Interacting MHC-I allele (IC_50_) on the nM scale
VTARAGLGY	HLA-A∗29:02 (40.62), HLA-A∗01:01 (224.16), HLA-A∗30:02 (168.45), HLA-B∗15:02 (81.70), HLA-C∗03:03 (103.66)
FHTVTVNTL	HLA-B∗39:01 (9.02), HLA-B∗38:01 (62.10), HLA-B∗15:02 (21.84), HLA-C∗03:03 (5.48), HLA-C∗08:02 (211.91), HLA-C∗14:02 (92.18), HLA-C∗12:03 (78.92)
HTTWTLTGY	HLA-A∗26:01 (162.41), HLA-A∗25:01 (219.32), HLA-B∗15:02 (153.54), HLA-C∗14:02 (122.92), HLA-C∗12:03 (33.90), HLA-C∗03:03 (144.75)
IELAGTLTL	HLA-E∗01:01 (152.83), HLA-B∗40:01 (35.47), HLA-B∗15:02 (55.75), HLA-C∗03:03 (29.28), HLA-C∗12:03 (109.70)
IASFSQPDW	HLA-B∗58:01 (9.34), HLA-B∗57:01 (42.06), HLA-B∗53:01 (61.22), HLA-C∗03:03 (2.35), HLA-C∗08:02 (187.57), HLA-C∗15:02 (164.60), HLA-C∗12:03 (11.95), HLA-C∗14:02 (248.10)
ARAGLGYQF	HLA-C∗12:03, HLA-C∗03:03 (53.53), HLA-B∗15:02 (107.95), HLA-B∗27:05 (97.18)
NYAWVNGNI	HLA-A∗68:02 (188.19), HLA-C∗14:02 (21.26), HLA-C∗03:03 (220.60), HLA-C∗12:03 (60.56)
KSYMSGNYK	HLA-C∗15:02 (6.94), HLA-A∗30:01 (9.18), HLA-A∗11:01 (11.28), HLA-A∗03:01 (20.22), HLA-A∗31:01 (54.63), HLA-C∗14:02 (40.80), HLA-C∗03:03 (61.75), HLA-C∗12:03 (31.71)
YMSGNYKAF	HLA-B∗15:01 (61.09), HLA-B∗35:01 (124.62), HLA-C∗14:02 (17.24), HLA-B∗15:02 (61.69), HLA-C∗03:03 (13.14), HLA-C∗12:03 (151.43)
SMYNTLWRV	HLA-A∗02:01 (6.70), HLA-A∗02:06 (13.79), HLA-C∗14:02 (105.59), HLA-C∗12:03 (30.21)
APKGSNKEI	HLA-B∗07:02 (199.48), HLA-C∗12:03 (7.82), HLA-C∗03:03 (37.81)

**Table 3:** The potential CD4^+^ T-cell epitopes along with their interacting MHC class II alleles with affinity (IC50) < 100 nM and respective predivac scores.

Epitope	Interacting MHC-II allele (IC_50_) on the nM scale	Number of alleles	Predivac score (binding core)
VTARAGLGYQFDLFA	HLA-DRB1∗04:01, HLA-DRB1∗09:01, HLA-DRB1∗04:05, HLA-DRB3∗01:01, HLA-DQA1∗05:01	5	68.76 (LGYQFDLFA)
NSGFHTVTVNTLDAT	HLA-DRB1∗01:01 (53), HLA-DRB1∗01:21 (17.87), HLA-DRB1∗01:17 (77.94), HLA-DRB1∗01:16 (98.93), HLA-DRB1∗01:13 (61.53), HLA-DRB1∗01:29 (83.08), HLA-DRB1∗01:24 (62.89), HLA-DRB1∗01:10 (28.18), HLA-DRB1∗01:11 (45.39), HLA-DRB1∗01:19 (39.99), HLA-DRB1∗01:12 (39.99), HLA-DRB1∗01:31 (39.99), HLA-DRB1∗01:32 (39.99), HLA-DRB1∗01:08 (39.99), HLA-DRB1∗01:05 (39.99), HLA-DRB1∗01:07 (39.99), HLA-DRB1∗01:27 (39.99), HLA-DRB1∗01:25 (39.99), HLA-DRB1∗01:22 (39.99), HLA-DRB1∗01:28 (39.99), HLA-DRB1∗01:14 (47.44), HLA-DRB1∗01:09 (37.12), HLA-DRB1∗01:15 (71.58), HLA-DRB1∗01:18 (44.25), HLA-DRB1∗01:06 (35.78), HLA-DRB1∗01:26 (38.4), HLA-DRB1∗01:20 (41.15), HLA-DRB1∗01:23 (48.69), HLA-DRB1∗01:04 (48.54), HLA-DRB1∗07:06 (65.25), HLA-DRB1∗07:05 (65.06), HLA-DRB5∗02:05 (46.61)	32	71.56 (FHTVTVNTL)
KAIELAGTLTLTGTP	HLA-DRB1∗01:01 (82), HLA-DRB1∗01:21 (23.85), HLA-DRB1∗01:17 (98.16), HLA-DRB1∗01:24 (58.9), HLA-DRB1∗01:10 (29.05), HLA-DRB1∗01:11 (49.8), HLA-DRB1∗01:19 (44.72), HLA-DRB1∗01:12 (44.72), HLA-DRB1∗01:31 (44.72), HLA-DRB1∗01:32 (44.72), HLA-DRB1∗01:08 (44.72), HLA-DRB1∗01:05 (44.72), HLA-DRB1∗01:07 (44.72), HLA-DRB1∗01:27 (44.72), HLA-DRB1∗01:25 (44.72), HLA-DRB1∗01:22 (44.72), HLA-DRB1∗01:28 (44.72), HLA-DRB1∗01:03 (91.98), HLA-DRB1∗01:14 (54.39), HLA-DRB1∗01:09 (31.87), HLA-DRB1∗01:15 (59.62), HLA-DRB1∗01:18 (40.48), HLA-DRB1∗01:06 (14.26), HLA-DRB1∗01:26 (18.43), HLA-DRB1∗01:20 (17.57), HLA-DRB1∗01:23 (24.27), HLA-DRB1∗01:04 (18.63), HLA-DRB1∗01:01 (82), HLA-DRB5∗02:05 (70.95)	29	71.70 (IELAGTLTL)
NNQIASFSQPDWESR		0	55.02 (FSQPDWESR)
VTARAGLGYQFDLFA		0	68.76 (LGYQFDLFA)
AQNYAWVNGNIKSDK	HLA-DRB5∗01:01 (62), HLA-DRB5∗02:04 (93.07), HLA-DRB5∗02:05 (24.55)	3	78.78 (YAWVNGNIK)
AAKSYMSGNYKAFLT	HLA-DRB1∗08:05 (65.65), HLA-DRB1∗12:03 (94.66), HLA-DRB1∗01:09 (57.66), HLA-DRB1∗01:10 (98.95), HLA-DRB1∗01:19 (99.23), HLA-DRB1∗01:12 (99.23), HLA-DRB1∗01:31 (99.23), HLA-DRB1∗01:32 (99.23), HLA-DRB1∗01:08 (99.23), HLA-DRB1∗01:05 (99.23), HLA-DRB1∗01:07 (99.23), HLA-DRB1∗01:27 (99.23), HLA-DRB1∗01:25 (99.23), HLA-DRB1∗01:22 (99.23), HLA-DRB1∗01:28 (99.23), HLA-DRB1∗01:21 (73.89), HLA-DRB1∗01:06 (52.24), HLA-DRB1∗01:23 (86.01), HLA-DRB1∗01:04 (87.14), HLA-DRB1∗01:26 (83.15), HLA-DRB1∗01:20 (85.84), HLA-DRB5∗02:05 (12.01), HLA-DRB5∗02:02 (67.2), HLA-DRB5∗02:04 (62.02)	24	60.30 (KSYMSGNYK)
SYMSGNYKAFLTEVN	HLA-DRB1∗01:21 (56.34), HLA-DRB1∗01:09 (89.52), HLA-DRB1∗01:10 (81.52), HLA-DRB1∗01:26 (98.98), HLA-DRB1∗01:06 (88.25), HLA-DRB1∗04:05 (38), HLA-DRB5∗02:05 (41.74)	7	69.98 (YKAFLTEVN)
ASMYNTLWRVNGQSA	HLA-DRB1∗08:05 (35.19), HLA-DRB1∗13:01 (49.45), HLA-DRB1∗12:05 (52.01), HLA-DRB1∗12:02 (60.35), HLA-DRB1∗12:03 (50.74), HLA-DRB1∗01:23 (62.08), HLA-DRB1∗01:26 (66.37), HLA-DRB1∗01:20 (74.19), HLA-DRB1∗01:06 (73.79), HLA-DRB5∗02:05 (44.37), HLA-DRB1∗11:01 (70)	11	82.45 (LWRVNGQSA)

**Table 4:** Conservancy analysis of all the epitopes with appropriate length.

Epitope	Conservancy	Length	Epitope	Conservancy	Length
VTARAGLGY	84.09%	9	**VTARAGLGY**QFDLFA	84.09%	15
SMYNTLWRV	100%	9	A**SMYNTLWRV**NGQSA	100%	15
KSYMSGNYK	97.73%	9	AA**KSYMSGNYK**AFLT	97.73%	15
NYAWVNGNI	100%	9	AQN**YAWVNGNI**KSDK	97.73%	15
HTTWTLTGY	97.73%	9	No prediction	Undetected	15
APKGSNKEI	100%	9	No prediction	Undetected	15
YMSGNYKAF	97.73%	9	S**YMSGNYKAF**LTEVN	97.73%	15
ARAGLGYQF	84.09%	9	VT**ARAGLGYQF**DLFA	84.09%	15
FHTVTVNTL	100%	9	NSG**FHTVTVNTL**DAT	100%	15
IELAGTLTL	100%	9	KA**IELAGTLTL**TGTP	100%	15
IASFSQPDW	97.73%	9	NNQ**IASFSQPDW**ESR	97.73%	15
YMSGNYKAF	97.73%	9	S**YMSGNYKAF**LTEVN	97.73%	15

## Abbreviations

SPATE: Serine protease autotransporter of enterobacteria
HEp-2: Human epithelial cell type-2
MHC-I: Major histocompatibility complex class I
MHC-II: Major histocompatibility complex class II
MSA: Multiple sequence alignment
GMMA: Generalized modules of membrane antigens
CTL: Cytotoxic T-lymphocyte
TAP: Transporter of antigenic peptide
SMM: Stabilized matrix method
HLA: Human leukocyte antigen.

## Additional Points

*Availability of Data and Materials*. Information about the data and their availability is intricately described in Methods.
