# Supplementary material for: Vaccinomics Approach for Designing Potential Peptide Vaccine by Targeting Shigella spp. Serine Protease Autotransporter Subfamily Protein SigA
Source: J Immunol Res. 2017 Sep 7;2017:6412353. doi: 10.1155/2017/6412353 (PMC5610819; doi:10.1155/2017/6412353)
Supplement: Supplementary file 1 — Table S1: Protein sequences retrieved from NCBI GenBank database for the analysis with appropriate accession numbers and antigenic score. Table S2: Population coverage analysis of the epitopes. Here the combined score for both MHC molecules have been represented. Table S3: The HLA I binding profile analyses of the selected peptides by EPISOPT. Figure S1: Dynamic interaction analysis of the clustered HLA alleles through tree illustration. Figure1A represents the MHC-I and Figure1B represents the MHC-II interaction. Here, HLA molecules are clustered on the basis of their preference for interaction with appropriate peptides. Figure S2: Ramachandran plot of the predicted model, which shows that most of the residues are in the allowed region of the plot, proving the validity of the model. Figure S3: Disorder prediction of the amino acid sequences of SigA. Here, our proposed epitopes localized outside of the disordered regions (15-35 and 520-530) and securing their potentiality for being an effective vaccine candidate. Notes: Amino acids in the input sequence are considered disordered when the blue line is above the gray dashed line, that is, when the confidence score is 0.5. The orange line shows the confidence score of the disordered protein-binding residue predictions. Figure S4: Control docking analysis of the epitope VMAPRTLIL and HLA-E allele. Figure S4A represents the oriented view of the interaction and assuring the perfect binding. Figure S4B represents the cartoon view and the Figure S4C embodies the interacted residues with the peptide. Figure S5: Variability plot for the 44 SigA protein alignment. The analysis was performed by using the PVS (protein Variability Software). The selected peptides were observed in the conserved region highlighted in the table. [file 6412353.f1.docx]

**Table S1:** Protein sequences retrieved from NCBI GenBank database for the analysis with appropriate accession numbers and antigenic score

| **Serial. No.** | **Entry name** |  | **Accession number** | **Organism** | **Antigenic Score** |
| --- | --- | --- | --- | --- | --- |
| 1 | Serine protease |  | gi\|446956854\| | *Enterobacteriaceae* | 0.6595 |
| 2 | Autotransporter |  | gi\|745759072\| | *Shigella flexneri* | *0.6592* |
| 3 | Autotransporter |  | gi\|647302223\| | *Shigella flexneri* | 0.6598 |
| 4 | Autotransporter |  | gi\|446956855\| | *Shigella sonnei* | 0.6591 |
| 5 | Autotransporter |  | gi\|446956852\| | *Shigella* | 0.6601 |
| 6 | Autotransporter |  | gi\|920402842\| | *Shigella sonnei* | 0.6590 |
| 7 | Autotransporter |  | gi\|920494373\| | *Shigella sonnei* | 0.6599 |
| 8 | Autotransporter |  | gi\|745770301\| | *Shigella flexneri* | 0.6634 |
| 9 | Autotransporter |  | gi\|920079095\| | *Shigella sonnei* | 0.6600 |
| 10 | Autotransporter |  | gi\|920393611\| | *Shigella sonnei* | 0.6587 |
| 11 | Autotransporter |  | gi\|920177380\| | *Shigella sonnei* | 0.6578 |
| 12 | Autotransporter |  | gi\|920409599\| | *Shigella sonnei* | 0.6628 |
| 13 | Autotransporter |  | gi\|920426430\| | *Shigella sonnei* | 0.6597 |
| 14 | Autotransporter |  | gi\|844758686\| | *Shigella boydii* | 0.6601 |
| 15 | Autotransporter |  | gi\|920049344\| | *Shigella sonnei* | 0.6577 |
| 16 | Autotransporter |  | gi\|916483308\| | *Shigella flexneri* | 0.6586 |
| 17 | Autotransporter |  | gi\|745773196\| | *Shigella boydii* | 0.6614 |
| 18 | Autotransporter |  | gi\|446956849\| | *Shigella* | 0.6627 |
| 19 | Autotransporter |  | gi\|446956853\| | *Shigella dysenteriae* | 0.6628 |
| 20 | Serine protease espP |  | gi\|391256698\| | *Shigella flexneri K-315* | 0.6583 |
| 21 | Autotransporter |  | gi\|920243191\| | *Shigella sonnei* | 0.6534 |
| 22 | Autotransporter |  | gi\|920548149\| | *Shigella sonnei* | 0.6605 |
| 23 | Serine protease EatA |  | gi\|903589225\| | *Shigella sonnei* | 0.6445 |
| 24 | Autotransporter |  | gi\|920451523\| | *Shigella sonnei* | 0.6606 |
| 25 | Autotransporter |  | gi\|920344021\| | *Shigella sonnei* | 0.6667 |
| 26 | Autotransporter |  | gi\|920402315\| | *Shigella sonnei* | 0.6620 |
| 27 | Autotransporter |  | gi\|920454114\| | *Shigella sonnei* | 0.6612 |
| 28 | Autotransporter |  | gi\|920465127\| | *Shigella sonnei* | 0.6551 |
| 29 | Serine protease EatA |  | gi\|903656641\| | *Shigella sonnei* | 0.6258 |
| 30 | Autotransporter |  | gi\|920469197\| | *Shigella sonnei* | 0.6642 |
| 31 | Serine protease EatA |  | gi\|905832519\| | *Shigella sonnei* | 0.6679 |
| 32 | Serine protease EatA |  | gi\|903886401\| | *Shigella sonnei* | 0.6241 |
| 33 | Serine protease EatA |  | gi\|903986032\| | *Shigella sonnei* | 0.6611 |
| 34 | Serine protease EatA |  | gi\|903905231\| | *Shigella sonnei* | 0.6368 |
| 35 | Serine protease EatA |  | gi\|903726783\| | *Shigella sonnei* | 0.6574 |
| 36 | Serine protease EatA |  | gi\|904265699\| | *Shigella sonnei* | 0.6473 |
| 37 | Autotransporter |  | gi\|920421786\| | *Shigella sonnei* | 0.6518 |
| 38 | Autotransporter |  | gi\|920430955\| | *Shigella sonnei* | 0.6581 |
| 39 | Autotransporter |  | gi\|920490928\| | *Shigella sonnei* | 0.6485 |
| 40 | Serine protease EatA |  | gi\|903849395\| | *Shigella sonnei* | 0.6569 |
| 41 | Serine protease EatA |  | gi\|903782758\| | *Shigella sonnei* | 0.6580 |
| 42 | Serine protease EatA |  | gi\|903532107\| | *Shigella sonnei* | 0.6611 |
| 43 | Serine protease EatA |  | gi\|906078334\| | *Shigella sonnei* | 0.6567 |
| 44 | Autotransporter |  | gi\|745767180\| | *Shigella sonnei* | 0.6699 |

**Table S2**: Population coverage analysis of the epitopes. Here the combined score for both MHC molecules have been represented.

| **Epitope (MHC-I)** | **Epitope (MHC-II)** | **Combined world coverage**  **(MHC I and MHC II)** |
| --- | --- | --- |
| FHTVTVNTL | NSGFHTVTVNTLDAT | 50.61% |
| NYAWVNGNI | AQNYAWVNGNIKSDK | 31.67% |
| KSYMSGNYK | AAKSYMSGNYKAFLT | 75.24% |
| YMSGNYKAF | SYMSGNYKAFLTEVN | 79.97% |
| IELAGTLTL | KAIELAGTLTLTGTP | **83.86%** |
| VTARAGLGY | VTARAGLGYQFDLFA | 63.88% |

**Table S3:** The HLA I binding profile analyses of the selected peptides by EPISOPT.

| **PEPTIDE** | **HLA I binding profile** |
| --- | --- |
| SMYNTLWRV | A0201 A0202 B3909 B4402 |
| NYAWVNGNI | A2402 A6802 B3801 |
| ARAGLGYQF | A6601 B2701 B2702 B2703 B2704 B2705 B2706 B2709 B39011 B3909 C0304 |
| IELAGTLTL | A0214 A2902 B1517 B4002 B4402 C0102 |
| VTARAGLGY | A1101 B1508 B1516 B5701 |
| KSYMSGNYK | A1101 |
| YMSGNYKAF | B1502 B4402 |
| FHTVTVNTL | B1509 B1510 B3801 B39011 B3909 |
| APKGSNKEI | B5101 B5102 B5103 B5401 B5502 |
| IASFSQPDW | B1513 |
| HTTWTLTGY |  |
